# Empowering Indigenous futures: advancing the 4Ms education model in American Indian and Alaska Native communities

**DOI:** 10.1093/geroni/igag104

**Published:** 2026-10-09

**Authors:** Barry J Wu, Megan Dicken, Ron Eppes, Jennifer Ouellet, Richard Marottoli

**Affiliations:** Department of Internal Medicine, Section of Geriatrics, Yale School of Medicine, New Haven, Connecticut, United States; International Association for Indigenous Aging (IA2), Silver Spring, Maryland, United States; International Association for Indigenous Aging (IA2), Silver Spring, Maryland, United States; Department of Internal Medicine, Section of Geriatrics, Yale School of Medicine, New Haven, Connecticut, United States; Department of Internal Medicine, Section of Geriatrics, Yale School of Medicine, New Haven, Connecticut, United States

**Keywords:** Age-Friendly, Education, Graphic, Indigenous

## Introduction

The United States (US) population of adults age 65 years and older has been increasing for decades. Between 2010 and 2020, this age group experienced its fastest growth since 1880–1890, increasing by 38.6% to over 55 million people.^1^ To address the need for caring for older adults, the John A. Hartford Foundation, Institute for Healthcare Improvement (IHI), American Hospital Association, Catholic Health Association of the United States, and leading geriatric care experts partnered in 2016 to develop a feasible and sustainable approach to making the US health care system age-friendly.^2^ Together, they created a framework called the 4Ms, which focuses on four essential elements of care for older adults: What Matters, Medication, Mentation, and Mobility. This 4Ms framework was introduced as part of the Age-Friendly Health System initiative with a goal to spread this model to 20% of US hospitals and health systems by 2020.^2^

Successful implementation of the Age-Friendly Health Systems initiative across the United States includes health systems serving American Indian and Alaska Native (AI/AN) peoples, such as the Indian Health Service (IHS), Tribal health systems, and Urban Indian clinics, which require culturally responsive adaptation to ensure relevance and effectiveness for the populations they serve. In 2024, the Health Resources and Services Administration (HRSA) provided funding under the Geriatrics Workforce Enhancement Program (GWEP) with the purpose of educating tribal organizations, underserved, and/or rural (TTOUR) primary care sites and delivery systems in the 4Ms framework.^3^ We describe the Yale COACH (Connecticut Older Adult Collaboration for Health) 4M (GWEP) partnership with the International Association for Indigenous Aging (IA2) and IHI in operationalizing a culturally responsive adaptation of the 4Ms framework specifically for AI/AN communities.

## Adapting 4Ms for AI/AN communities

We sought to adapt the language and visual imagery associated with the 4Ms to be more relatable and relevant to AI/AN communities. Listening sessions with AI/AN tribal members and elder service providers from 10 states and IA2 constituents highlighted the need to adapt the 4Ms in a form that is familiar to all Native nations and represents strength, connection, and rootedness in Indigenous values. Through an iterative process of refinement and assistance from a graphic designer, the image progressed from an original IHI 4Ms graphic of four concentric circles around one large circle to a medicine wheel with four segments and pictures representing seasons of the year to a tree with a trunk, three branches, roots, and leaves (Figure 1). The graphic was further refined following presentations at the 2025 California Indian Manpower Consortium (CIMC) Caregiver Training, 2025 National Indian Council on Aging (NICOA) American Indian Elder conferences, and input and approval from IHI representatives. This work was presented as a poster at the 2025 Gerontological Society of America meeting.^4^

**Figure 1:**
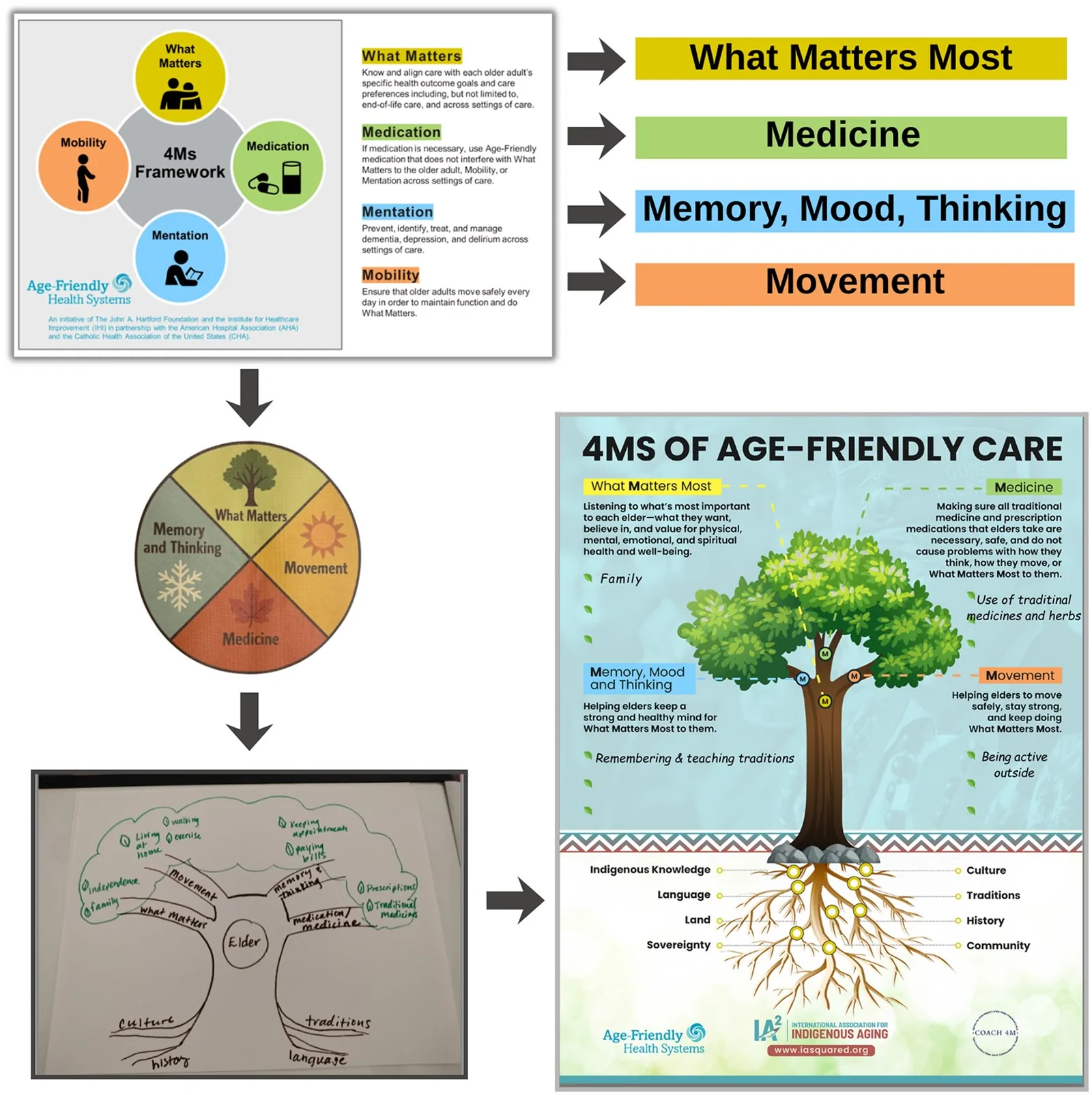
4Ms Innovation for American Indian and Alaska Native communities.

## The tree model

The 4Ms graphic for AI/AN communities incorporates the cultural elements of a tree with a trunk and three branches representing the 4Ms. The trunk of the tree is “What Matters Most,” highlighting the importance and centrality of family in AI/AN communities. The roots were added to the tree and labeled with what is universally important to Indigenous people, including Indigenous knowledge, language, land, sovereignty, culture, traditions, history, and community. For each of the 4Ms, a leaf provided more relatable language such as family, use of traditional medicine and herbs, being active outside, and remembering and teaching tradition. Moreover, the language for the 4Ms was modified to replace medical jargon with terms AI/AN communities could identify with: “Medications” changed to “Medicine”, which included traditional medicines and herbs, “Mobility” to “Movement,” and “Mentation” to “Memory, Mood and Thinking”. Additionally, “Elder” is a respected term in AI/AN communities and preferred to “Older Adult”.

## Discussion

The 4Ms framework was introduced as a part of the Age-Friendly Health Systems initiative in the late 2010s to promote high-quality care for older adults. It has been adopted by thousands of hospitals and health care systems nationwide and embedded in the electronic health care record. Education to AI/AN communities, however, has been limited. Working in partnership with AI/AN communities, elders, providers, researchers, and IHI, IA2 and the Yale COACH 4M team developed a culturally responsive adaptation of the 4Ms framework, incorporating community-reflective imagery and language. The addition of leaves was an innovation to provide examples and enhance communication. This innovative graphic framework and adapted terms can be further modified to reflect local tribal geographic elements, language, and traditions unique to the region. It also enhances the two-way communication between patients and families with their providers. As the population is aging not only in the United States, but globally, it is essential to operationalize innovation in the field of gerontology by adapting and listening to what is most important to the populations we serve.

## Data Availability

Materials are available and can be accessed by contacting the authors. This study was not preregistered.
